# Impact of robust treatment planning on single- and multi-field optimized plans for proton beam therapy of unilateral head and neck target volumes

**DOI:** 10.1186/s13014-017-0931-8

**Published:** 2017-11-28

**Authors:** Macarena Cubillos-Mesías, Michael Baumann, Esther G. C. Troost, Fabian Lohaus, Steffen Löck, Christian Richter, Kristin Stützer

**Affiliations:** 1 OncoRay – National Center for Radiation Research in Oncology, Faculty of Medicine and University Hospital Carl Gustav Carus, Technische Universität Dresden, Helmholtz-Zentrum Dresden – Rossendorf, Dresden, Germany; 2 Department of Radiotherapy and Radiation Oncology, Faculty of Medicine and University Hospital Carl Gustav Carus, Technische Universität Dresden, Dresden, Germany; 3 German Cancer Consortium (DKTK), partner site Dresden, and German Cancer Research Center (DKFZ), Heidelberg, Germany; 4 Helmholtz-Zentrum Dresden - Rossendorf, Institute of Radiooncology – OncoRay, Dresden, Germany; 5 National Center for Tumor Diseases (NCT), partner site Dresden, Dresden, Germany

**Keywords:** Proton therapy, Single-field optimization, Multi-field optimization, Robust optimization, Adaptive radiation therapy, Head and neck cancer

## Abstract

**Background:**

Proton beam therapy is promising for the treatment of head and neck cancer (HNC), but it is sensitive to uncertainties in patient positioning and particle range. Studies have shown that the planning target volume (PTV) concept may not be sufficient to ensure robustness of the target coverage. A few planning studies have considered irradiation of unilateral HNC targets with protons, but they have only taken into account the dose on the nominal plan, without considering anatomy changes occurring during the treatment course.

**Methods:**

Four pencil beam scanning (PBS) proton therapy plans were calculated for 8 HNC patients with unilateral target volumes: single-field (SFO) and multi-field optimized (MFO) plans, either using the PTV concept or clinical target volume (CTV)-based robust optimization. The dose was recalculated on computed tomography (CT) scans acquired during the treatment course. Doses to target volumes and organs at risk (OARs) were compared for the nominal plans, cumulative doses considering anatomical changes, and additional setup and range errors in each fraction. If required, the treatment plan was adapted, and the dose was compared with the non-adapted plan.

**Results:**

All nominal plans fulfilled the clinical specifications for target coverage, but significantly higher doses on the ipsilateral parotid gland were found for both SFO approaches. MFO PTV-based plans had the lowest robustness against range and setup errors. During the treatment course, the influence of the anatomical variation on the dose has shown to be patient specific, mostly independent of the chosen planning approach. Nine plans in four patients required adaptation, which led to a significant improvement of the target coverage and a slight reduction in the OAR dose in comparison to the cumulative dose without adaptation.

**Conclusions:**

The use of robust MFO optimization is recommended for ensuring plan robustness and reduced doses in the ipsilateral parotid gland. Anatomical changes occurring during the treatment course might degrade the target coverage and increase the dose in the OARs, independent of the chosen planning approach. For some patients, a plan adaptation may be required.

**Electronic supplementary material:**

The online version of this article (10.1186/s13014-017-0931-8) contains supplementary material, which is available to authorized users.

## Background

Radiation therapy for head and neck cancer (HNC) is one of the pillars of treatment, being a definitive (possibly combined with chemotherapy) or adjuvant (after surgery, possibly combined with chemotherapy) treatment option. Due to the complexity of the target volumes and the surrounding organs at risk (OAR), the treatment planning process is particularly challenging. In general, radiation therapy is delivered using high-energy photons. However, nowadays particle beam therapy, in particular proton beam irradiation, is increasingly being introduced in clinical practice [1]. Intensity modulated proton therapy (IMPT) was shown to be promising for the treatment of HNC in terms of high dose conformity, with reduced dose to the normal tissue in comparison with photon therapy [2–5].

Due to the physical characteristics of dose deposition, protons are more sensitive to uncertainties than photons. These uncertainties can arise from changes in the patient anatomy throughout the treatment course, by e.g. tumor shrinkage, different cavity filling or weight loss, from daily variations in patient setup, from uncertainties in the proton range due to the conversion of computed tomography (CT) numbers to stopping power ratios [6, 7] and due to uncertainties in the beam delivery system. The optimization method of pencil beam scanning (PBS) proton therapy plans can be single-field (SFO) or multi-field optimization (MFO). In SFO, the spot positions and weights of each proton field are optimized individually, so the resultant dose distribution by each field is uniform over the target volume. In MFO, the spots from all the fields are optimized together, generating highly conformal dose distributions. Unlike SFO, the dose from individual MFO fields can be relatively inhomogeneous [8, 9].

Previous studies have shown that the conventional planning target volume (PTV) concept is not sufficient for providing robustness in target coverage in proton therapy [6, 10, 11], although SFO plans plus PTV margins could provide enough robustness [8]. Different optimization methods have been investigated to optimize plans which are robust against setup and range uncertainties [12–14], and some of them are already available in commercial treatment planning systems (TPS). Such robust plans are generated based on the clinical target volume (CTV) and take certain scenarios of setup errors and proton range uncertainties into account.

Unilateral HNC targets are an indication for proton beam therapy in our institution in case of re-irradiation or subtypes of salivary tumors, such as adenoidcystic carcinoma. Although unilateral HNC targets might not be as challenging as bilateral targets, the chosen beam direction and configuration, which is patient dependent, as well as different dose distribution and proximity to organs at risk, can influence the plan robustness and might indicate a different strategy for robust planning. For example, van der Voort et al. [15] have shown that unilateral cases seem to be intrinsically less robust than bilateral cases using a minimax worst-case scenario optimization. Different PBS proton planning studies can be found in literature for bilateral HNC targets, but only a few of them consider unilateral targets [16–18]. However, in these studies the authors have only taken the dose distribution on the initial planning CT into account without considering anatomical changes that may occur during the treatment course. The aim of this study was therefore to compare SFO and MFO PBS proton plans in combination with robust optimization for unilateral HNC targets in terms of, e.g. target coverage, dose reduction to the OARs, and to assess the robustness to range and setup uncertainties as well as to anatomical changes.

## Methods

### Patient data

Eight subsequent patients with unilateral head and neck tumors, treated with double scattering proton therapy at the University Proton Therapy Dresden were selected (Table 1). Each dataset consisted of a planning CT (pCT) and several control CTs (cCT) (median: 6, range: 3–13) acquired during the course of treatment using an in-room dual energy CT on-rails. For both, planning and cCTs, pseudo-monoenergetic CT data sets (79 keV) were reconstructed from dual-energy CT scans (80 kVp/140 kVp) [7].

**Table 1:** Patient characteristics, volume of CTVs and details on imaging for verification

Patient	Primary tumor site	Gender	Clinical/pathological TNM-stage	No. of cCTs	CTV / cm^3^			
					Low-risk		High-risk	
1	Mucoepidermoid carcinoma of left parotid gland	F	pT1 N0 M0	6	159.7	(150.5–158.7)	17.1	(15.5–17.8)
2	Adenoidcystic carcinoma of right parotid gland	M	pT1 N0 M0	13	92.3	(87.7–96.3)	44.5	(42.3–46.4)
3	Small salivary glands of oral cavity	M	pT1 N2b M0	5	218.2	(199.9–211.2)	55.9	(50.3–53.3)
4	Lateral border of tongue	F	pT1 N2b M0	11	79.8	(79.0–83.9)	16.0	(16.2–17.1)
5	Right tonsil	M	pT2 N2b M0	11	150.2	(136.2–157.0)	43.2	(39.2–45.5)
6	Maxillary sinus	M	rcT2 N1 M0	5	134.9	(130.3–141.5)	28.8	(24.2–27.7)
7	Adenoidcystic carcinoma of left parotid gland	M	pT4a N0 M0	3	246.0	(238.4–242.6)	93.7	(87.9–91.7)
8	Recurrent adenoidcystic carcinoma of right submandibular gland	M	rpT2 N0 M0	6	78.7	(78.1–81.9)	22.5	(23.1–24.0)

Abbreviations: *F* Female, *M* Male, *cCT* Control CTs, *CTV* Clinical target volume

CTV values: pCT (range in cCTs)

CTVs and OARs spinal cord, brainstem, parotid gland, larynx, oral mucosa, pharyngeal constrictor muscles and esophageal inlet muscle were contoured on the pCT by an experienced radiation oncologist. A high-risk CTV including primary tumor, surgical cavity and potentially metastatic lymph nodes was expanded by the elective lymph nodes to generate a low-risk CTV. The pCT was registered with each cCT, the contours were transferred using a deformable registration algorithm [19], reviewed and if necessary corrected by the same radiation oncologist.

### Treatment planning

The PTVs were generated for non-robust optimized plans by isotropic expansion of the CTVs by 5 mm to account for setup and range uncertainties [11]. The prescribed doses were 50.3 Gy(RBE) to the low-risk and 68 Gy(RBE) to the high-risk CTV, delivered by simultaneous integrated boost (SIB) in 34 fractions.

Four PBS proton plans were generated for each patient:

- Conventional SFO and MFO plans with PTV concept, i.e. using the PTVs as target volumes (SFO_PTV_, MFO_PTV_).
- Robust optimized SFO and MFO plans (SFO_Rob_, MFO_Rob_), using the CTVs as target volumes and accounting 3 mm for setup uncertainty and 3.5% for range uncertainty, as used in our institution, considering in total 21 different scenarios in the minimax approach [6, 13]. Objective functions related to the target volumes were selected for robust optimization [20].

The plans were calculated and analyzed in RayStation v4.99 (RaySearch Laboratories AB, Stockholm, Sweden), considering a relative biological effectiveness (RBE) of 1.1. Two or three beams were used with the same configuration for the four different plans, avoiding entering through risk structures and inhomogeneity regions, with gantry angles between 15°-40° for the first beam, 70°-80° for the second beam and 160°-170° for the third beam, with couch rotations between 0°-20°. An IBA universal nozzle beam, with a pencil beam spot size sigma ranging from 8 mm (100 MeV) to 4 mm (220 MeV) was used for the calculations. The spot distance was calculated automatically by the TPS. Moreover, a calculation dose grid of 3x3x3 mm^3^ and a range shifter of 7.5 cm water equivalent thickness were considered. An additional transitional intermediate volume between low-risk and high-risk region of 10 mm margin was created [15] for the SIB dose gradient.

The four plans were optimized to deliver the prescribed dose to the target volumes (*D*_98%_ ≥ 95% and *D*_2%_ ≤ 107% of the prescribed dose, where *D*_98%_ and *D*_2%_ are the minimum doses to 98% and 2% of the target volume, respectively), while sparing the clinical OARs following the institutional protocol: spinal cord: maximum dose (*D*_max_) < 45 Gy, brainstem: *D*_max_ < 54 Gy and parotid gland: median dose (*D*_median_) ≤ 26 Gy. The remaining OARs were considered for dose reporting.

### Nominal plan robustness against setup and range uncertainty

**Fig. 1:**
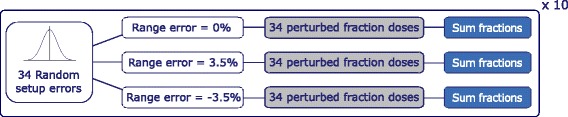
Generation of 30 randomly perturbed dose distributions for the whole treatment course of 34 fractions

For evaluating the plan robustness on the pCT, perturbed doses with random setup uncertainties and fixed range uncertainty values of −3.5%, 0% and 3.5% were generated. For each treatment fraction, a random number was drawn from a Gaussian distribution with mean μ = 0 mm and standard deviation σ = 2.5 mm [21, 22] for the isocenter shift in each cardinal direction (x, y, z). For each range uncertainty (−3.5% / 0% / 3.5%), 34 single-fraction doses with different random setup uncertainties were calculated on the pCT and summed to generate a new perturbed integral-treatment dose, which takes into account the random variation of the setup error in each fraction. This procedure was repeated 10 times for each range uncertainty scenario as displayed in Fig. 1, resulting in 30 cumulated perturbed doses considering only the anatomy from the pCT (${\overline{D}}_{\mathrm{pCT}}$). The dose-volume histograms (DVH) from the 30 results were plotted as DVH bands [23] for visual comparison.

### Doses on control CTs

The four nominal plans per patient were recalculated in each cCT, in order to evaluate the influence of the anatomical changes on the dose distributions. For comparing the approximated delivered dose with the nominal plan, cumulative doses (*D*_cCT_) were calculated by deforming [19] and summing the fraction doses from the cCTs on the pCT.

The plan robustness against setup and range errors including the anatomical changes was evaluated following the procedure as for the nominal plan. Again, 30 cumulative perturbed doses (${\overline{D}}_{\mathrm{cCT}}$) were generated, each with the same range error and 34 random setup errors, but using the different cCTs for the respective fraction dose calculations.

### Plan adaptation

An adaptive planning strategy was applied to compensate for anatomy-related discrepancies between nominal and fraction doses. Each fraction dose recalculated in the cCTs was compared with the nominal plan, considering the *D*_98%_ for low- and high-risk CTV and *D*_2%_ for high-risk CTV as dose parameters. Whenever a worsening of >5 percentage points between planned and unperturbed fraction dose was found [24], an adapted plan using the cCT of the respective fraction was calculated. Same beam configurations and objective functions as in the original plan were used.

A cumulative dose (*D*_cCT, Adapt_) was calculated as the sum of the fraction doses from the initial plan before adaptation plus the fraction doses with adapted plan. For practical purposes, the adaptation was considered to be initiated at the third fraction after the control CT with insufficient dose parameters to simulate a clinical scenario with required QA procedures for the new plan. This new adapted cumulative dose *D*_cCT, Adapt_ was compared with the cumulative doses without adaptation *D*_cCT_.

### Statistical analysis

One-way analysis of variance (ANOVA) followed by post-hoc two-sample independent *t*-tests were performed in SPSS (IBM Corporation, New York, USA) to find significant differences in dose parameters between the four nominal plans, including Bonferroni correction for multiple testing. Paired *t*-tests were used to determine significant differences between the planned dose *D*_pCT_, the cumulative dose *D*_cCT_, and the adapted cumulative dose *D*_cCT, Adapt_. Two-sided tests were performed and *p*-values <0.05 were considered to be significant.

## Results

### Evaluation of nominal plans

**Fig. 2:**
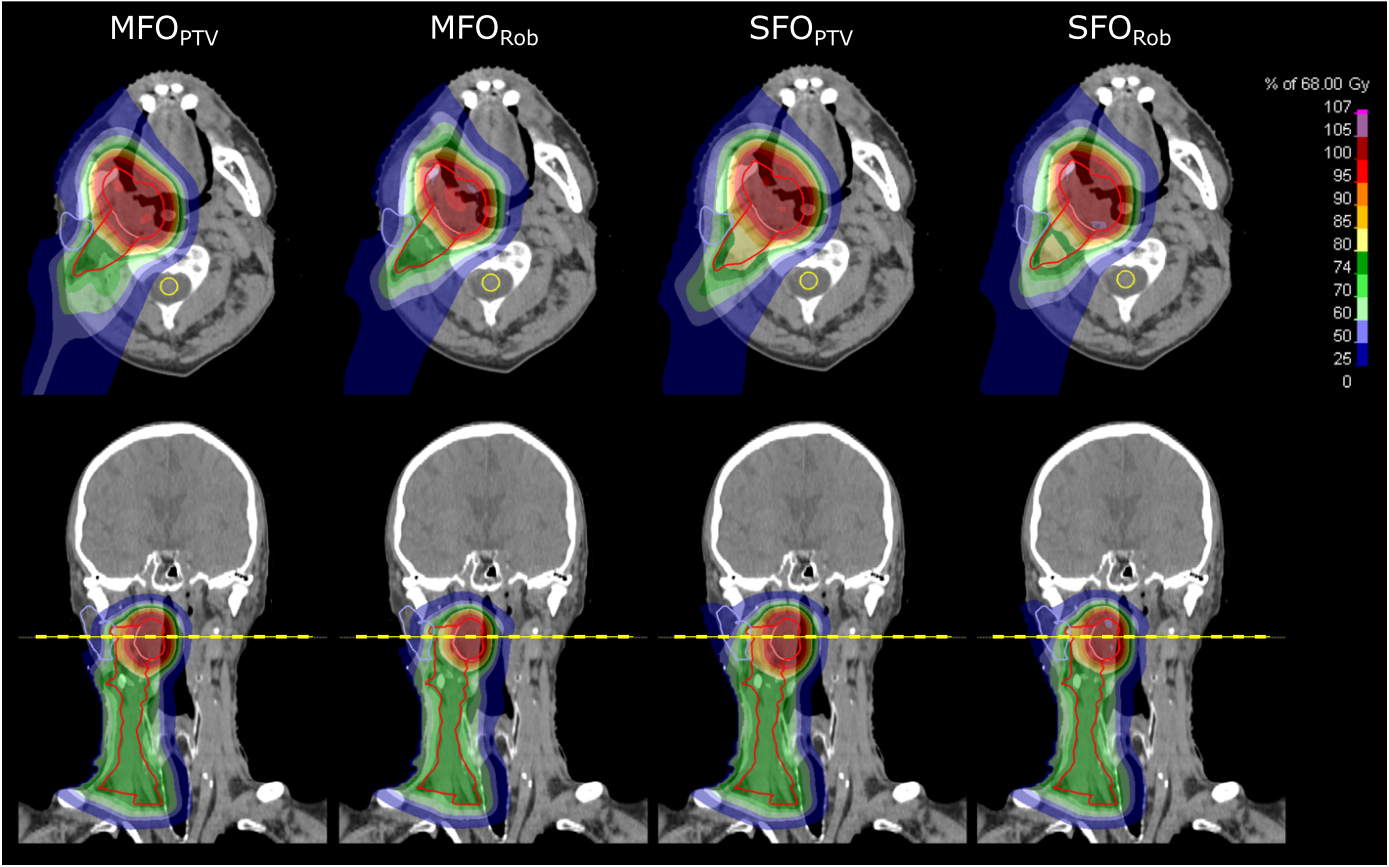
Dose distributions for an example patient, in transversal (top) and coronal (bottom) view. The high-risk CTV is contoured in light red, the low-risk CTV in dark red, the ipsilateral parotid gland in light blue and the spinal cord (in the transversal view only) in yellow. The yellow dashed line indicates the location of the transversal view

**Fig. 3:**
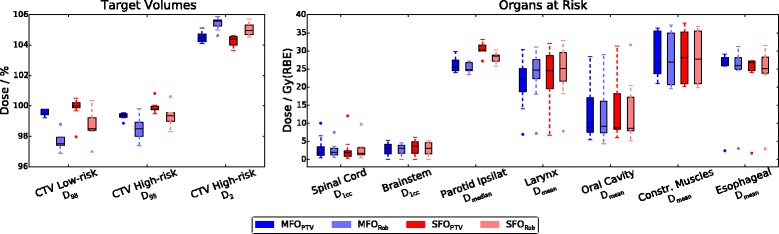
Dose statistics from the 8 patients for target volumes (left) and organs at risk (right) in the nominal plans

Dose distributions for an exemplary patient are shown in Fig. 2. For all patients, target coverage was similar for the four plans, fulfilling the specification (*D*_98%_: 96.9–100.5% for the low-risk CTV, 97.4–100.8% for the high-risk CTV), but being slightly lower for the robust optimized plans, as displayed in Fig. 3. Regarding hot spots, *D*_2%_ ≤ 107% was met by the four plans in the high-risk CTV (103.6–106.3%). *D*_2_ values higher than 107% can be found in the low-risk CTV due to the dose gradient for the SIB treatment, and were therefore not evaluated.

**Fig. 4:**
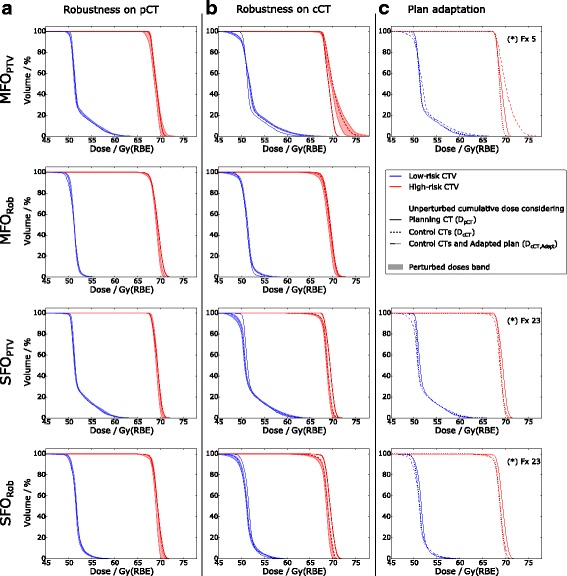
DVHs from the four planning approaches MFO_PTV_, MFO_Rob_, SFO_PTV_ and SFO_Rob_ (top to bottom) for the low- (blue) and high-risk CTV (red) of an example patient who received plan adaptation for 3 plans after the 5th and 23th fraction. The DVH from the nominal plan (solid line, shown in all plots for orientation) is complemented by the DVH bands from the 30 perturbed cumulative doses for robustness analysis considering the initial plan and (**a**) the nominal anatomy (${\overline{D}}_{pCT}$), and (**b**) anatomy in the control CTs (${\overline{D}}_{cCT}$). DVH from the unperturbed cumulative dose considering the anatomical changes and additional plan adaptation (**c**) are indicated by dashed and dotted lines. (*) indicates the fraction number of the control CT when the adaptation demand was found

The doses to the OARs were similar for all planning strategies (Fig. 3). The near-maximum doses to the spinal cord (*D*_1cc_: 0.36–12.05 Gy(RBE)) and brainstem (*D*_1cc_: 0.01–6.17 Gy(RBE)) were far below the clinical constraints. For the ipsilateral parotid (only contoured in five patients due to surgical removal in the others), higher median doses were found in both SFO approaches. This dose increase was statistically significant for the SFO_PTV_ plans (ANOVA: *p* = 0.005; MFO_PTV_ vs. SFO_PTV_: *p* = 0.026, MFO_Rob_ vs. SFO_PTV_: *p* = 0.006). The contralateral parotid was completely spared in all cases (*D*_median_: 0-0.1 Gy(RBE)). For the mean dose in the larynx, oral mucosa, constrictor muscles and esophageal inlet muscle, the doses between the four planning approaches were similar. Integral doses to the normal tissue [25] presented no differences between the different plans (mean values averaged over patient cohort: 36.47–37.84 Gy • Liter, standard deviation: 9.70–10.79 Gy • Liter). The 30 perturbed cumulated doses ${\overline{D}}_{\mathrm{pCT}}$ resulted in wider DVH bands for the CTVs in the case of MFO_PTV_ plans, while the other 3 planning approaches showed a smaller and comparable band width (Fig. 4a). A high dose tail in the low-risk CTV can be observed in the PTV approaches, due to the PTV margin expansion used for both CTVs. An additional patient example can be found in Additional File 1: Fig. S1a, possessing wider DVH bands for both CTVs.

### Evaluation of control CTs

The differences between the cumulative doses considering the anatomical variations in the control CTs (*D*_cCT_) with the initial anatomy (*D*_pCT_) are presented in Table 2. Comparing the differences *D*_cCT_ − *D*_pCT_, high discrepancies on the target doses were observed for the MFO_PTV_ plan in one case, in which the hotspot dose in the high-risk CTV was increased by up to 5.5%.However, when analyzing the whole patient cohort, these variations were not statistically significant, compared to the other three plan approaches. The *D*_98%_ showed no significant differences in the low-risk CTV, whereas for the high-risk CTV the SFO_Rob_ plans revealed a significant *D*_98%_ reduction in the *D*_cCT_ of 0.78% (*p* = 0.012). Regarding the OARs, individual cases presented higher cumulative doses than the planned ones (e.g. up to 8 Gy in larynx *D*_mean_ for MFO_PTV_ and SFO_PTV_ plans in one patient). Table 3 shows target volume changes from the pCT to the last cCT. For some patients, volume shrinkage in both targets are observed (e.g. patient 3 and 5), whereas for other patients slight increase was found (e.g. patient 4 and 6).

**Table 2:** Median (range) of differences between the cumulated dose considering the anatomy in the control CTs, *D*_*cCT*_, and the nominal plan dose *D*_*pCT*_. A positive number indicates a higher value of *D*_*cCT*_. Significant differences (paired *t*-test, *p* < 0.05) are marked by*

ROI Parameter	MFO_PTV_	MFO_Rob_	SFO_PTV_	SFO_Rob_
Low-risk CTV				
ΔD_98%_ / pp	−0.09	0.16	0	0.05
	(−3.62–0.58)	(−0.58–0.58)	(−4.14–0.76)	(−2.70–0.32)
High-risk CTV				
ΔD_98%_ / pp	0.15	−0.53	−0.18	−0.78*
	(−1.03–1.94)	(−3.41–0.16)	(−2.50–0.16)	(−2.65 − −0.21)
ΔD_2%_ / pp	0.18	−0.31*	−0.46*	−0.63*
	(−0.29–5.51)	(−0.88 − −0.06)	(−0.91 − −0.13)	(−0.94 − −0.34)
Spinal Cord				
ΔD_1cc_ / Gy	0.57	0.61	0.46	0.59*
	(−1.21–1.15)	(−0.82–1.04)	(−0.72–0.99)	(−0.80–1.22)
Brainstem				
ΔD_1cc_ / Gy	0.61	0.13	0.15	0.17
	(−0.82–1.04)	(−0.14–1.80)	(−0.24–1.14)	(−0.17–1.39)
Parotid Ipsilateral				
ΔD_median_ / Gy	0.66	0.04	−0.31	−0.34
	(−0.02–2.39)	(−1.66–1.78)	(−2.02–1.34)	(−2.66–1.34)
Larynx				
ΔD_mean_ / Gy	1.21	0.89	1.21	0.9
	(−1.37–8.58)	(−1.28–6.60)	(−1.51–8.86)	(−1.29–6.97)
Oral Mucosa				
ΔD_mean_ / Gy	−0.05	0.02	0.01	0.06
	(−0.48–1.79)	(−0.20–1.61)	(−0.72–1.72)	(−0.71–1.54)
Constrictor Muscles				
ΔD_mean_ / Gy	0.1	−0.11	0.05	0.05
	(−2.05–2.83)	(−2.09–2.35)	(−2.45–4.16)	(−2.24–2.91)
Esophageal inlet				
ΔD_mean_ / Gy	0.57	0.44	0.57	0.5
	(−1.67–7.84)	(−1.20–5.23)	(−1.64–8.15)	(−1.28–5.53)

Abbreviations: *pp.* Percentage points, *D*_*98%*_ Minimum dose to the 98% of the target volume, *D*_*2%*_ Minimum dose to the 2% of the target volume, *D*_*mean*_ Mean dose, *D*_*median*_ Median dose

**Table 3:** Difference in cm^3^ between target volume in planning CT and last control CT. Positive (negative) values correspond to volume reduction (increase). Patients who underwent plan adaptation are marked by*

Patient	Volume change (cm^3^)	
	Low-risk CTV	High-risk CTV
1	8.11	−0.03
2*	3.98	2.19
3*	18.25	5.54
4	−1.97	−0.94
5*	7.79	2.27
6*	−6.60	4.46
7	7.61	5.76
8	−3.23	−1.41

When evaluating the robustness against setup and range errors while considering the anatomical changes (${\overline{D}}_{\mathrm{cCT}}$), again the MFO_PTV_ plans show wider bands in the CTV region as can be seen in Fig. 4b for the high-risk CTV. An additional patient example can be found in Additional File 1: Fig. S1b, possessing wider DVH bands for both CTVs.

### Plan adaptation evaluation

Nine plans from 4 patients required plan adaptation, as summarized in Table 4. Four PTV-based plans and 5 robustly optimized plans needed adaptation.

**Table 4:** Overview of adapted plans, the respective adaptation criteria and the fraction number of the control CT in which the adaptation demand was found

Patient	Plan	Adaptation criteria	Fraction
2	MFO_Rob_	ΔD_98%_ (low-risk CTV)	24
	SFO_Rob_	ΔD_98%_ (low-risk CTV)	24
3	MFO_PTV_	ΔD_2%_ (high-risk CTV)	6
	SFO_PTV_	ΔD_98%_ (low-risk CTV)	23
	SFO_Rob_	ΔD_98%_ (low-risk CTV)	23
5	MFO_PTV_	ΔD_2%_ (high-risk CTV)	17
6	MFO_Rob_	ΔD_98%_ (high-risk CTV)	25
	SFO_PTV_	ΔD_98%_ (high-risk CTV)	25
	SFO_Rob_	ΔD_98%_ (high-risk CTV)	25

Table 5 compares for the 9 adapted cases the differences between the nominal plan *D*_pCT_, the cumulative dose *D*_cCT_ and the cumulative adapted dose *D*_cCT, Adapt_. The target coverage in the cumulative dose was improved after adaptation (*p* = 0.013 for *D*_98%_(low-risk CTV), *p* = 0.017 for *D*_98%_(high-risk CTV)). The mean dose to the larynx was reduced in the adapted plans, whereas the other OARs showed no major improvement, except in individual cases (e.g. mean dose of the esophageal inlet muscle reduced by 9.6 Gy for patient 3 in the adapted MFO_PTV_ plan). Fig. 4c includes DVHs of *D*_cCT, Adapt_ for an example patient.

**Table 5:** Median (range) of the dosimetric difference between the estimated delivered dose by the nominal (*D*_*pCT*_), the cumulative (*D*_*cCT*_) and adapted plan (*D*_*cCT*, *Adapt*_) for the investigated 9 cases. A positive number indicates a higher value for *D*_*cCT*_. Significant differences (paired *t*-test, *p* < 0.05) are marked by *

ROI Parameter	*D*_cCT_ − *D*_pCT_	*D*_cCT_ − *D*_cCT, Adapt_
CTV Low-risk		
ΔD_98%_ / pp	−0.76*	−0.52*
	(−4.14–0.42)	(−2.76 – −0.14)
CTV High-risk		
ΔD_98%_ / pp	−1.15*	−0.6*
	(−3.41–0.25)	(−2.22–0.25)
ΔD_2%_ / pp	−0.34	0.37
	(−0.94–5.51)	(−0.13–6.74)
Spinal Cord		
ΔD_1cc_ / Gy	0.43	−0.15
	(−0.82–1.14)	(−0.22–0.79)
Brainstem		
ΔD_1cc_ / Gy	0.74*	0.22
	(−0.24–1.8)	(−0.19–1.07)
Parotid Ipsilateral		
ΔD_median_ / Gy	0	−0.26
	(−2.66–1.78)	(−1.16–0.73)
Larynx		
ΔD_mean_ / Gy	6.39*	1.69*
	(−1.51–8.86)	(−0.87–9.05)
Oral Mucosa		
ΔD_mean_ / Gy	−0.2	−0.23
	(−0.72–1.79)	(−0.66–2.29)
Constrictor Muscles		
ΔD_mean_ / Gy	0.66	0.22
	(−2.45–4.16)	(−0.86–4.26)
Esophagus		
ΔD_mean_ / Gy	0	0.13
	(−1.64–8.15)	(−0.93–9.61)

Abbreviations: *pp.* Percentage points, *D*_*98%*_ Minimum dose to the 98% of the target volume, *D*_*2%*_ Minimum dose to the 2% of the target volume, *D*_*mean*_ Mean dose, *D*_*median*_ Median dose

## Discussion

We compared four different PBS proton therapy approaches for HNC patients with unilateral target volumes, SFO and MFO with and without robust optimization. Furthermore, we studied the influence of anatomical changes on the dose distributions, and applied plan adaptation when needed.

The PTV-based non-robust and the CTV-based robust approaches fulfilled the target coverage requirements in the nominal plan. The near maximum doses to spinal cord and brainstem were below the clinical constrains due to the unilateral target location, which allowed for an increased dose sparing compared to bilateral targets. However, both SFO plans gave significantly higher median doses to the ipsilateral parotid than the MFO approaches. This can be explained by the reduced degrees of freedom in the field modulation in comparison with MFO, an issue already investigated for bilateral HNC targets [8]. The contralateral parotid gland was completely spared due to the chosen beam configuration, which reduces the risk of severe xerostomia [26]. The plan optimization did not focus on dose to the larynx, oral mucosa, constrictor muscles and esophageal inlet, but dose values for these organs were similar for the different planning approaches and the DVH values were close to or below the recommended constraints for reducing the risk of larynx edema and dysphagia [27]. The 5 mm PTV margin expansion was chosen from our experience in photon therapy. We hesitated to reduce this margin for non-robust planning. If we would choose a smaller margin, the median doses in the ipsilateral parotid gland could be reduced. However, as already mentioned, due to the field modulation in SFO the parotid doses might still exceed the clinical objectives.

The MFO_PTV_ plans showed reduced robustness against uncertainties, while the other 3 approaches provided sufficient robustness on target coverage. Due to their characteristics in dose deposition, MFO_PTV_ plans are more sensitive to uncertainties in both patient setup and proton range than SFO_PTV_ and robustly optimized plans. Therefore they bear the risk to deliver a dose inferior to the prescribed one to the targets and OARs (see Additional File 2: Fig. S1). Beam direction and configuration might also influence the robustness of the plans. We chose two or three beams simulating a realistic clinical scenario. Some studies have found no significant differences in CTV coverage and plan robustness when the beam number is increased for bilateral HNC targets [28, 29].

When considering the anatomical changes during the treatment course, for individual cases, it was found that a lower dose than the planned dose might be delivered to the CTVs. These differences were independent of the planning approach, showing that both SFO and MFO techniques are sensitive to anatomical changes, being the same for PTV-based and robust approaches. The same conclusion can be drawn for the dose to the OARs, where the highest dose changes were found for PTV-based plans, especially for the larynx, constrictor muscles and esophagus inlet muscle. Furthermore, the target volume changes and the need of adaptation were patient dependent: patient 3 presented in both targets volume shrinkage, whereas for patient 6, which also underwent into adaptation, presented a slight increase of the low-risk CTV and a moderate shrinkage of the high-risk CTV. For a detailed conclusion, the size of the patient cohort is not sufficient. When considering a worst case scenario including anatomical changes (see Additional File 2: Fig. S2), the doses to the OARs could be highly increased, and the target coverage reduced as well, in comparison to a worst case scenario only considering setup and range uncertainties. The influence of anatomical changes could not be sufficiently estimated by the plan robustness evaluation in the planning CT, which explains why both PTV-based and robust-based plans underwent adaptation with a comparable frequency.

In 7 of the 9 cases with plan adaptation, the delivery of the adapted plan started within the last 10 fractions of the treatment. The adaptation was necessary due to an underdosage in the low- or high-risk CTV, except for two MFO_PTV_ plans, where the criterion was an overdose in the CTV high-risk. The cumulated dose from the adapted plans *D*_cCT, Adapt_ improved the target coverage, but they did not improve the OAR dose in comparison to the cumulative doses without adaptation *D*_cCT_, except for the larynx. The quite small, but partially significant improvements can be found because in most cases the number of fractions with adapted plan was small, but the cumulative doses for the whole treatment were considered for the analysis. For bilateral HNC targets, Kurz et al. [30] did not find a significant difference in the target coverage between the recalculated dose in the control CT and the adapted plan, but the adaptation showed a reduction of overdosage (D_2%_) in the target volume. However, Góra et al. [31] found a large improvement in the target coverage with the generation of adapted MFO_PTV_ plans on a biweekly basis, whereas the OAR dose limits were not exceeded, even without adaptation. The difference between both studies might be explained because Kurz et al. optimized their adapted plan in a cone-beam CT (CBCT)-based virtual CT. It is known that proton dose calculations on CBCT-based images have limitations in accuracy due to several reasons [32, 33]. Hence, the CBCT-based adaptation might therefore not be as optimal as an adaptation planned directly on a control CT, as done by Góra et al. Besides, a strong case-dependency in bilateral HNC targets could be responsible for non-consistent results in the literature. In this work, the adaptation procedure was applied when a difference of >5 percentage points between planned and a single fraction dose was found. Probably in some cases the adaptation might also be necessary if many consecutive fractions show inferior target coverage, although each difference with the planned dose remains lower than 5 percentage points. Adaptation criteria considering the deviations of the tracked accumulated dose from the planned dose may be able to detect a reduction in the target coverage earlier than comparing it with the individual fraction doses. In the present study, only dose deviations in the target volumes were considered, but variations in the OARs dose parameters during the treatment course should be taken into account as well.

All the evaluations including calculation of SFO and MFO plans in combination with robust optimization, dose calculation in additional CTs, dose deformation and dose accumulation were performed in a commercial TPS, which means that the evaluation tools could be easily translated into clinical routine.

## Conclusions

The four PBS planning approaches showed adequate target coverage on the nominal plan for unilateral HNC. For ensuring plan robustness and reduced median doses in the ipsilateral parotid gland, MFO_Rob_ is recommended. Both PTV-based and robust optimized plans were sensitive to anatomical changes for individual cases, leading to inferior target coverage and higher OARs dose, and requiring plan adaptation. None of the 4 planning approaches presented a clear superiority concerning the need of plan adaptation.

## Additional files

Additional file 1: Dose-volume histograms for additional patient example. (PDF 156 kb)

Additional file 2: Worst case analysis. (PDF 423 kb)

## Abbreviations

*D*_cCT, Adapt_: Cumulative adapted dose
*D*_cCT_: Cumulative dose
*D*_pCT_: Nominal dose
${\overline{D}}_{cCT}$: Perturbed cumulative dose
${\overline{D}}_{pCT}$: Perturbed dose on the pCT
ANOVA: Analysis of variance
CBCT: Cone-beam CT
cCT: Control CT
CT: Computer tomography
CTV: clinical target volume
*D*_2%_: Minimum dose to the 2% of the target volume
*D*_98%_: Minimum dose to the 98% of the target volume
*D*_mean_: Mean dose
*D*_median_: Median dose
DVH: Dose-volume histogram
HNC: Head and neck cancer
IMPT: Intensity modulated proton therapy
MFO: Multi-field optimization
MFO_PTV_: MFO PTV-based plan
MFO_Rob_: MFO robust-optimized plan
OAR: Organ at risk
PBS: Pencil beam scanning
pCT: Planning CT
pp: Percentage points
PTV: Planning target volume
RBE: Relative biological effectiveness
ROI: Region of interest
SFO: Single-field optimization
SFO_PTV_: SFO PTV-based plan
SFO_Rob_: SFO robust-optimized plan
SIB: Simultaneous integrated boost
TPS: Treatment planning system
