# Supplementary material for: Impact of robust treatment planning on single- and multi-field optimized plans for proton beam therapy of unilateral head and neck target volumes
Source: Radiat Oncol. 2017 Nov 28;12:190. doi: 10.1186/s13014-017-0931-8 (PMC5706329; doi:10.1186/s13014-017-0931-8)
Supplement: Supplementary file 1 — Dose-volume histograms for additional patient example. (PDF 156 kb) [file 13014_2017_931_MOESM1_ESM.pdf]

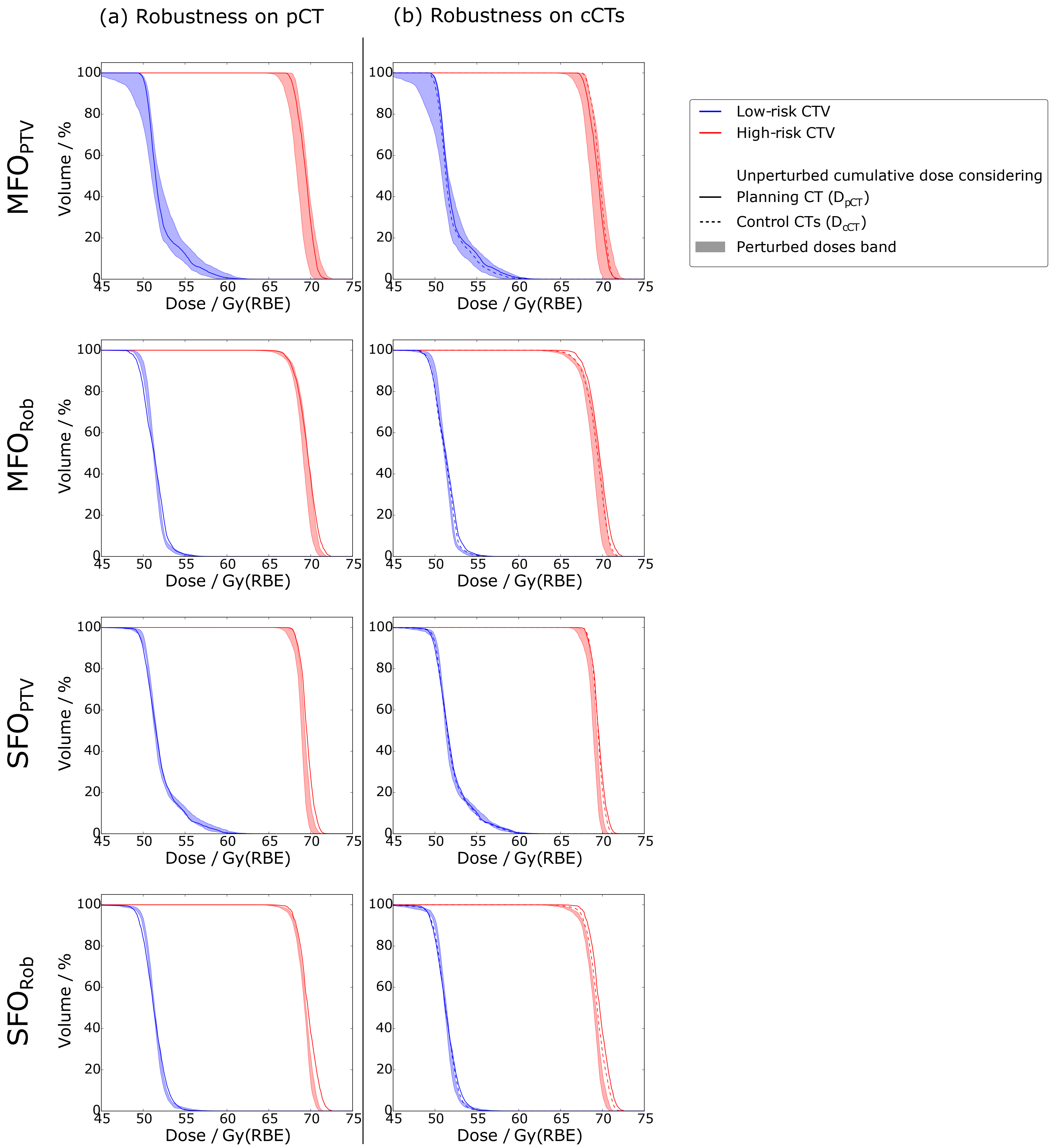

Figure S1. DVHs from the four planning approaches MFO$_{PTV}$, MFO$_{Rob}$, SFO$_{PTV}$ and SFO$_{Rob}$ (top to bottom) for the low- (blue) and high-risk CTV (red) of an example patient. The DVH from the nominal plan (solid line, shown in all plots for orientation) is complemented by the DVH bands from the 30 perturbed cumulative doses for robustness analysis considering the initial plan and (a) the nominal anatomy ($\bar{D}_{pCT}$), and (b) anatomy in the control CTs ($\bar{D}_{cCT}$).
