# Supplementary material for: Impact of robust treatment planning on single- and multi-field optimized plans for proton beam therapy of unilateral head and neck target volumes
Source: Radiat Oncol. 2017 Nov 28;12:190. doi: 10.1186/s13014-017-0931-8 (PMC5706329; doi:10.1186/s13014-017-0931-8)
Supplement: Supplementary file 2 — Worst case analysis. (PDF 423 kb) [file 13014_2017_931_MOESM2_ESM.pdf]

Additional File 2

Worst case analysis

The worst case values were taken from the 30 cumulative perturbed doses from the robustness analysis, calculated either in the planning CT ($\overline{D}_{\mathrm{pCT}}$, figure S1), or considering the anatomy changes in each fraction ($\overline{D}_{\mathrm{cCT}}$, figure S2). They are represented by the minimum of the 30 cumulative perturbed doses for the D_{98} in both CTVs and the maximum for the D_2 in the high-risk CTV and mean/median doses in the organs at risk. These values where compared with the nominal doses.

The worst case values obtained with our approach come from a likely cumulative dose distribution that could be delivered in the presence of fraction random setup uncertainties and range uncertainties, instead of considering a single fraction scenario [1, 2], when the worst case values are overestimated.

References

1. Liu W, Frank SJ, Li X, Li Y, Zhu RX, Mohan R. PTV-based IMPT optimization incorporating planning risk volumes vs robust optimization. Med Phys. 2013;40:021709. doi:10.1118/1.4774363.

2. Barten DLJ, Tol JP, Dahele M, Slotman BJ, Verbakel WFAR. Comparison of organ-at-risk sparing and plan robustness for spot-scanning proton therapy and volumetric modulated arc photon therapy in head-and-neck cancer. Med Phys. 2015;42:6589–98. doi:10.1118/1.4933245.

The worst cases values were taken from the 30 perturbed doses with random setup uncertainties and fixed range uncertainties calculated either in the planning CT($\bar{D}_{\text{pCT}}$), or considering the anatomy changes in each fraction ($\bar{D}_{\text{cCT}}$).

The lowest value for D_{98}, and the highest value for D_2 and OARs were taken from the perturbed doses and then compared with the nominal plan values.

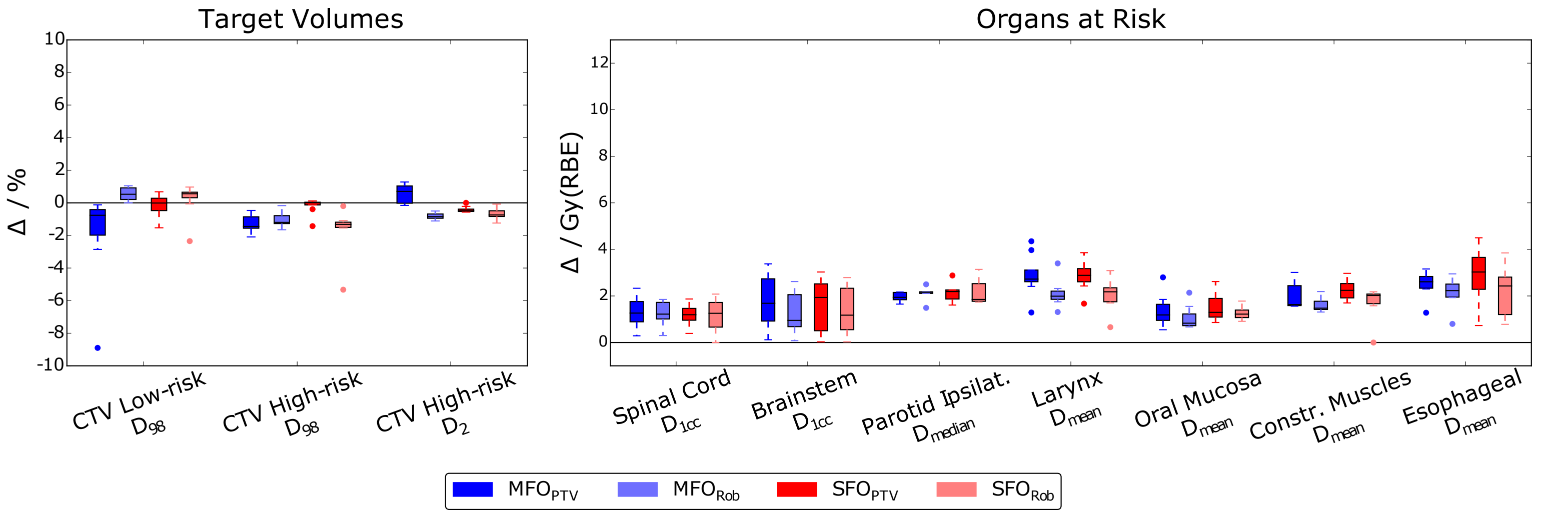

Figure S2. Difference between the worst case values of the 30 perturbed doses $\bar{D}_{\text{pCT}}$ and the nominal plan values D_{pCT}. Positive values indicate higher dose on the worst case.

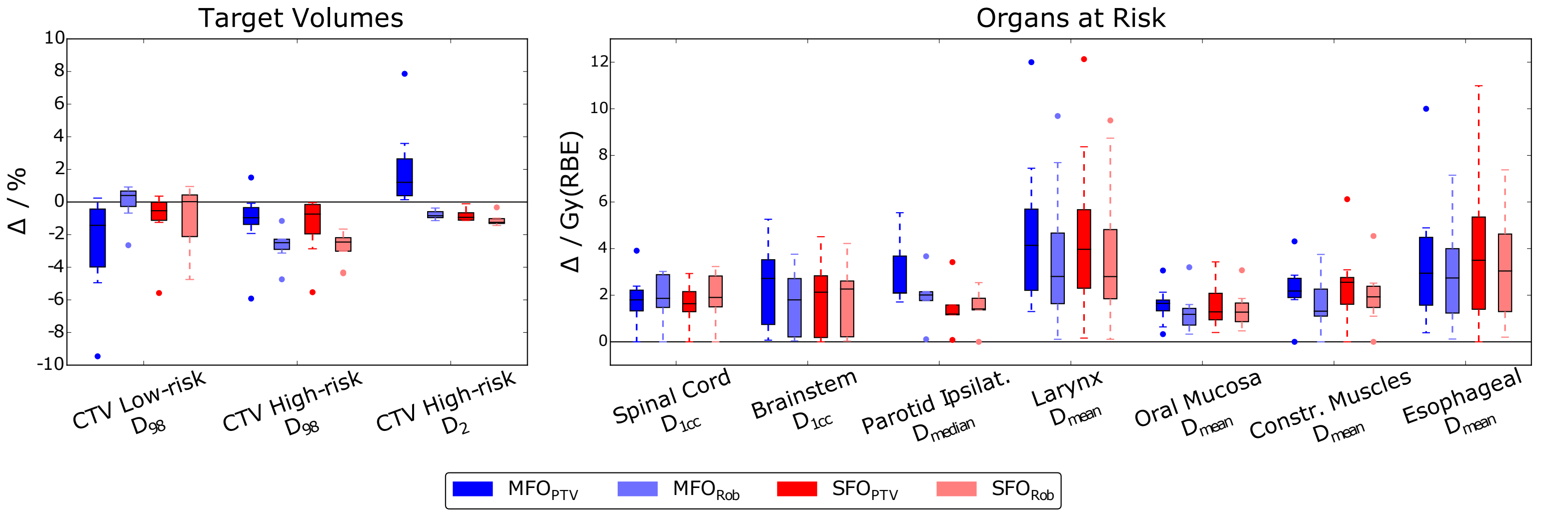

Figure S3. Difference between the worst case values of the 30 perturbed doses considering anatomical changes $\bar{D}_{\text{cCT}}$ and the nominal plan values D_{pCT}. Positive values indicate higher dose on the worst case.
